# Pharmacological Treatment of Major Depressive Disorder in Adolescents

**DOI:** 10.1100/tsw.2005.55

**Published:** 2005-05-13

**Authors:** Rachel L. Farley

**Affiliations:** Columbia University College of Physicians and Surgeons, New York, USA

**Keywords:** adolescence, depression, pharmacotherapy, United States

## Abstract

Major depressive disorder (MDD) affects a significant number of adolescents today. Its consequences (including social isolation, failure to achieve crucial developmental milestones, and suicide) mandate close attention in clinical practice. While tricyclics and monoamine oxidase inhibitors (MAOIs) have been used infrequently and with questionable efficacy, selective serotonin reuptake inhibitors (SSRIs), particularly fluoxetine, consistently have been shown to be of benefit in treating outpatient adolescents with MDD. Despite some success with other drugs in its class, fluoxetine remains the only SSRI that is FDA approved for treatment of children and adolescents with depression. A review of recent studies is presented, including the controversy regarding the relationship of antidepressants and suicidal behavior in this patient population.

## INTRODUCTION

During adolescence, milestones of growth, maturation, and adaptation often interact to result in an experience consisting of various difficulties and frustrations. For many people, a salient feature of adolescence is the experience of depression. As such, it is the responsibility of many figures involved (including teachers, counselors, parents, and physicians) to recognize the common patterns of depression in adolescents. Primary care physicians, in particular, are in a unique position to identify signs, diagnose, intervene, and potentially treat depression in their patients.

## AN OVERVIEW OF DEPRESSION IN ADOLESCENCE

The experience of depression, or depressive symptoms, in the adolescent population is not an uncommon phenomenon. The prevalence of depression in adolescents is reported in some series to be up to 8.3% with lifetime prevalence rates as high as 15–20%[1]. Prior to onset of puberty, rates of depression appear to be equal between boys and girls. However, this pattern changes dramatically with puberty and subsequent maturation into adulthood. As such, adolescent girls are two to three times more likely to experience depression than boys of the same age. Incidentally, this ratio is also observed among adult patients with depression. Furthermore, the ways in which adolescent patients manifest symptoms of depression differ largely from their adult counterparts. Somatic complaints, behavioral problems, anxiety, and phobias are frequently seen in younger patients, whereas functional impairment and actual sadness and gloom are exhibited largely by adult patients[1]. In particular, children and adolescents with major depressive disorder commonly appear irritable and may even be aggressive on account of their sense of frustration and irritability[2].

The significant morbidity and mortality associated with depression in adolescents is cause for concern and an impetus to improve the likelihood of diagnosis. Among the consequences of untreated depression is a failure to achieve the tasks associated with adolescent development, such as gaining independence from one's family and parental figures, achieving self-identity, and forming meaningful interpersonal relationships[3]. Even more devastating is the increased probability of experiencing major depression as an adult and the heightened risk of suicide in depressed adolescents. The recurrence rates for major depressive disorder (MDD) are reported to be 70% within a 5-year period[1]. Suicide accounts for 12% of the mortality in adolescents, making it one of the leading causes of death in this age group[1]. In fact, more than half of adolescents and children with MDD will make at least one suicide attempt. Depressed adolescents have a 14-fold increase in lifetime suicide attempts, when compared to nondepressed peers[4].

These consequences, both immediate and in the future of a patient, make prompt diagnosis and treatment of depression in adolescents imperative. There are a number of treatment options available to the clinician, including various forms of psychotherapy, medical management alone, or a combination of both approaches. Despite the reported successes of psychotherapy (cognitive-based therapy in particular) in adolescents with depression or depressive symptoms[5], a substantial emphasis has been placed on research into the pharmacologic treatment of depression in this patient population based on successful outcomes in adults. A number of classes of antidepressant drugs exist and have been studied in adolescents with often-conflicting results and even surprising side effects.

## TRICYCLIC ANTIDEPRESSANTS

Tricyclic antidepressants, including amitryptiline, imipramine, desipramine, nortriptyline, and doxepin, have been prescribed for depression in adults for decades. In adolescent and pediatric patients, virtually no randomized controlled trials have demonstrated efficacy compared to placebo to date. Placebo response rates have varied and been reported as high as 70%[6]. Given this high placebo response, it may be difficult to ascertain the true effect of tricyclics in these studies. Furthermore, controlled trials have been underpowered in having small sample sizes, ranging from 20–45 adolescents in both the treatment and placebo groups combined[6]. There have been numerous reasons suggested for the consistently poor treatment responses cited in studies of tricyclic antidepressants, despite prior efficacy in adults. Some examples include neurobiological differences in adult vs. childhood or adolescent depression, immaturity of neurotransmitter systems, and possible comorbid diagnoses that may interfere with successful treatment of depression per se[7]. In addition to these factors, the side-effect profile of tricyclics is less than ideal, particularly for younger patients. Cardiotoxicity and anticholinergic properties account for the most serious of these adverse effects, and thereby limit use both in patients with family history of related problems (i.e., cardiac disease) and in patients unable to tolerate such symptoms[6]. Additional effects of tricyclics can be attributed to peripheral noradrenergic and cholinergic blockade, resulting in orthostatic hypotension, dry mouth, constipation, and confusion[8]. For these reasons, tricyclics are prescribed infrequently for both children and adolescents and are not considered first-line therapies for either group.

## MONOAMINE OXIDASE INHIBITORS (MAOI)

Similarly, monoamine oxidase inhibitors (phenelzine, tranylcypromine, isocarboxazid, selegiline, and pargyline) are primarily used in adults, albeit less commonly than in past decades due to development of newer antidepressants. MAOIs also carry generally unfavorable side-effect profiles including, but not limited to, dietary restriction of tyramine-containing foods, numerous drug interactions, headaches, and dizziness, and further have a narrow therapeutic index, making overdose a potentially fatal event[9]. While few studies conducted in the 1980s demonstrated a possible effect in adolescent patients[10], the lack of randomized controlled trials testing the effects of MAOIs in adolescents and children is perhaps a testament to the infrequent use of this class of antidepressants in these age groups.

## SELECTIVE SEROTONIN REUPTAKE INHIBITORS (SSRI)

SSRIs such as fluoxetine, sertraline, paroxetine, fluvoxamine, and citalopram have been used in the treatment of MDD as well as a number of other psychiatric diagnoses such as generalized anxiety disorder and obsessive-compulsive disorder since their introduction in 1987. In 1997, a randomized controlled trial of fluoxetine vs. placebo in both children and adolescents aged 7–17 years revealed a statistically significant antidepressant effect of the drug as measured by improvement on Clinical Global Impression-Severity scale, or CGI-S, over an 8-week treatment period (56% response rate vs. 33% in the placebo group). Scores on the Children's Depression Rating Scale-Revised (CDRS-R) similarly improved within each group. Despite this outcome, complete remission of depressive symptoms occurred in less than one-third of patients in the fluoxetine group and less than one-quarter of patients in the placebo group[11]. A confirmatory study involving a larger sample size of both children and adolescents was published 5 years later in 2002, indicating once again the superiority of fluoxetine over placebo in the acute-phase treatment of MDD using the same measures in this patient population. The rate of clinical remission in the fluoxetine group (41%) was more than twice that of the placebo group (20%). Based on these results, the authors concluded that daily fluoxetine therapy was a "well tolerated and effective treatment for depression in children and adolescents"[12].

A more recently published randomized trial examined the efficacy of fluoxetine in children and adolescents for preventing relapse of depression. The authors supplemented the findings of the previous 2002 trial by following responders in both the fluoxetine and placebo groups for a "relapse-prevention phase" consisting of an additional 32 weeks in which patients in the treatment group were again randomized to either fluoxetine or placebo (responders in the original placebo group continued to receive the inactive drug). In the patients initially treated with fluoxetine in the acute phase, the mean time to relapse, as defined by CDRS-R scores, was more than twice as long in the fluoxetine group than the placebo group. Additionally, 60% of the placebo group met criteria for relapse within the study period, compared to only 34% in the treatment group. At the end point, the fluoxetine group had significantly lower mean Clinical Global Impression-Improvement scores (CGI-I) than the placebo group. The majority of relapses in the group treated with placebo (following discontinuation of fluoxetine for the acute phase) occurred within 8 weeks. Thus, it was concluded that fluoxetine is an effective treatment not only for the acute phase of major depressive disorder in children and adolescents, as initially demonstrated, but also for relapse prevention in the months following clinical remission[13].

To expand on these significant findings regarding usage of fluoxetine in the adolescent populations, the Treatment for Adolescents with Depression Study (TADS) compared the effects of treatment with fluoxetine alone, cognitive-based therapy (CBT) alone, fluoxetine with CBT, and placebo on a final sample size of 351 adolescents between the ages of 12 and 17 years. The same outcome measures (CDRS-R and CGI-I) were used. In this study, only the fluoxetine and placebo were administered double blind, whereas the groups with CBT alone and with fluoxetine were not blinded. A number of comparisons were made among the treatment groups, yielding surprising results. Across the 12-week treatment period, CBT with fluoxetine was significantly superior to placebo, fluoxetine alone, and CBT alone. Fluoxetine alone and CBT alone were not found to differ significantly from the effects of placebo; however, fluoxetine alone was superior to CBT alone. Effect sizes of each treatment group decreased in order with fluoxetine with CBT as the highest (0.98), fluoxetine was second highest (0.68), and CBT was the lowest (and was actually found to have a slightly negative effect size of –0.03). As such, CBT alone was found to be statistically similar to placebo. With respect to suicidality, defined specifically as suicidal ideation, fluoxetine with CBT was the only treatment group found to be superior to placebo statistically. Again, both fluoxetine alone and CBT alone were not found to differ statistically from placebo on this measure. Additionally, fluoxetine alone was not superior to CBT alone, and the effect size of CBT alone actually surpassed that of fluoxetine with CBT and fluoxetine alone, indicating a possible protective effect of CBT in the setting of suicidality[14].

Similar findings have been reported in studies of other SSRIs in the adolescent population. Randomized controlled trials of sertraline in 376 children and adolescents with moderate to severe depression in several countries have demonstrated greater improvements in CDRS-R scores when compared to placebo throughout the 10-week course of treatment. Specifically, the difference between groups was approximately 10% in each of the clinical measures of improvement used. While this difference was not considered statistically significant, the authors maintained the beneficial impact of these results based on the larger and more diverse sample population in this study than in prior studies of any SSRI[15]. Another controlled trial of paroxetine in 275 adolescents aged 12–18 years also incorporated the tricyclic antidepressant, imipramine, into the study design to demonstrate superiority of SSRIs over placebo as well as other drug classes. The results of this study confirmed the lack of response to tricyclics when compared to placebo response rates, as had been reported earlier in the literature. The larger sample size of the group randomized to imipramine (95 adolescents) lends further support to this finding, as small sample sizes in the past had been counted among the possible causes of the negative results for tricyclics. Paroxetine was significantly superior to placebo on most clinical parameters utilized. These included total score and depressed mood item on Hamilton Rating Scale for Depression (or HAM-D) and depressed mood item on the Schedule for Affective Disorders and Schizophrenia for Adolescents-Lifetime version (or K-SADS-L). CGI score improvement and overall response were also significantly higher in the paroxetine group. However, despite comparisons made with each against placebo, there was no direct comparison of paroxetine and imipramine to demonstrate superiority conclusively[16]. Citalopram has also been examined in this patient population. A randomized controlled trial of children and adolescents with depression showed a 36% response rate in the citalopram-treated subjects and a 24% placebo response rate after 8 weeks. Superiority of citalopram was demonstrated as early as 1 week as well as consistently throughout this period, as measured by change in CDRS-R scores[17].

Thus, randomized controlled trials of multiple SSRIs have demonstrated efficacy and superiority over placebo. Despite the promising results in sertraline, citalopram, and paroxetine, the most consistent and convincing response rates have been associated with treatment with fluoxetine. Whether fluoxetine is superior to others in its class, or its earlier introduction has contributed to the substantial literature on its efficacy, remains to be decided. It is likely that further research efforts with the other SSRIs will yield similarly consistent and supportive results.

The safety profiles of SSRIs have been considered favorable in comparison to the alternatives, tricylics and MAOIs. Certainly, no pharmacologic agent is without adverse effects. Fluoxetine is considered the safest in its class for adolescents and children based on its higher risk-benefit profile as indicated by numerous studies demonstrating both efficacy and low side-effect rates[18]. Nonetheless, side effects reported in all SSRIs include nausea, vomiting, diarrhea, dermatitis, change in appetite, weight change, nervousness, anxiety, headache, manic episodes, insomnia, drowsiness, and sexual dysfunction. The serotonin syndrome has been reported in the setting of increased dosage of or initial treatment with SSRIs. Unlike older classes of antidepressants, cardiac and neurotoxicities are exceptionally rare, and lethal overdose is much less likely based on lower toxicity[8,10].

Recent concerns have been raised, based largely on case reports, regarding the risk of suicidality in children and adolescents treated with SSRIs. While the true relationship is not yet defined, there are known risk factors for suicide and suicidal attempts: a prior suicidal attempt or previous suicidal behavior and having been prescribed more than one antidepressant in the past and/or over time[19]. Whether the causal relationship in question is due to the effect of an antidepressant, or perhaps related to the severity of disease (in which case antidepressants are often prescribed for more severe cases of depression) is not yet known. An analysis of suicide rates reported trials testing effects of fluoxetine, sertraline, paroxetine, citalopram, and other non-SSRI antidepressants across treatment and placebo groups found no significant difference in absolute suicide rates and rates by cumulative exposure across any of these groups (SSRI, other antidepressant, and placebo). In fact, other antidepressants (including nefazodone, mirtazepine, bupropion, maprotiline, trazodone, mianserin, dothiepin, imipramine, amitryptiline, and venlafaxine) were associated collectively with a slightly higher rate of patient suicides, although this finding was not statistically significant[20]. On the other hand, a review of randomized controlled trials of SSRIs indicate that although these antidepressants decrease suicidality in some depressed patients, a larger number of suicidal acts (attempts and completions) has been documented in patients receiving active treatment as compared to placebo[21]. The data are clearly mixed on this issue. Until a more definitive correlation becomes available, all antidepressants have been given a "black box warning" by the FDA regarding the possibility of suicidal behavior in pediatric patients. Despite warnings of potential dangers associated with certain pharmacologic agents, providers should carefully assess the risk-benefit ratio of treatments and take into account the potential for suicidal behavior in the absence of treatment in the setting of depression.

## OTHER ANTIDEPRESSANTS

Although the data are limited, literature has been published recently on newer antidepressants in children and adolescents that do not conform to the aforementioned drug classes. An open-label study reports on the efficacy of mirtazepine, a drug that enhances both serotonergic and noradrenergic transmission, in 23 adolescents in an open-label trial lasting 12 weeks. The authors reported a 78.3% response rate measured by a 50% decrease in HAM-D-17 total score and a 60.9% rate of reduction of Beck Depression Inventory scores by 50%. Additionally, 87% of the patients had "some improvement" on the CGI-I scale. Common adverse effects included dizziness, drowsiness, and increased appetite[22]. Other newer antidepressants studied in children and adolescents include venlafaxine, in which no statistically significant difference in response vs. placebo was demonstrated in a 6-week protocol[23], as well as bupropion, which demonstrated potential efficacy in eight adolescents in an open trial despite significant side effects (insomnia and weight loss)[24]. Because a number of non-SSRI antidepressants have been successful in treatment of adults, one might expect further research on these agents in younger populations in the future.

## CONCLUSIONS

Fluoxetine is currently considered the safest of the SSRIs for the treatment of depression in children and adolescents. This may be due to the greater volume of literature on fluoxetine, as compared to sertraline, paroxetine, citalopram, and other SSRIs. The combination of fluoxetine and CBT has been shown to be more efficacious than either treatment alone in managing adolescent depression. Although similar literature incorporating psychotherapy with other SSRIs has not yet been published, it is reasonable for the provider to determine the appropriate SSRI and/or psychotherapy-based treatment according to an assessment of patient needs, cost, compliance with therapy, time restrictions, and clinician availability. The American Academy of Child and Adolescent Psychiatry has created guidelines for the management of MDD and other affective disorders in this population. Currently, recommendations for the acute phase of treatment include both psychotherapy (i.e., interpersonal therapy, CBT, psychodynamic psychotherapy) and pharmacotherapy in the setting of bipolar and psychotic depression, severe symptomatic depression, and failure to respond to psychotherapy. Among the various pharmacologic treatments, SSRIs are the medication of choice[25], again reflecting its proven efficacy and safety.

In summary, there are many options available to the physician or therapist to initiate treatment of MDD in the adolescent patient. While no one method has been associated with a 100% response rate, many have demonstrated substantial effect sizes in randomized controlled trials, the gold standard for assessing drug efficacy. Moreover, results of noncontrolled clinical trials have been promising and will likely stimulate further research in controlled settings.

## BIOSKETCH

**Rachel L. Farley, BA,** is a medical student at Columbia University College of Physicians and Surgeons. E-mail: rlf2017@columbia.edu
